# Biodegradation and metabolic pathway of sulfamethoxazole by *Sphingobacterium mizutaii*

**DOI:** 10.1038/s41598-021-02404-x

**Published:** 2021-11-30

**Authors:** Jinlong Song, Guijie Hao, Lu Liu, Hongyu Zhang, Dongxue Zhao, Xingyang Li, Zhen Yang, Jinhua Xu, Zhiyong Ruan, Yingchun Mu

**Affiliations:** 1 Key Laboratory of Control of Quality and Safety for Aquatic Products (Ministry of Agriculture and Rural Affairs), Chinese Academy of Fishery Sciences, Beijing, 100141 China; 2 Key Laboratory of Healthy Freshwater Aquaculture, Ministry of Agriculture and Rural Affairs, Key Laboratory of Freshwater Aquaculture Genetic and Breeding of Zhejiang Province, Zhejiang Institute of Freshwater Fisheries, Huzhou, 313001 China; 3 College of Food Science and Engineering, Bohai University, Jinzhou, 121013 China; 4 Institute of Agricultural Resources and Regional Planning, CAAS, Beijing, 100081 China

**Keywords:** Environmental biotechnology, Environmental microbiology

## Abstract

Sulfamethoxazole (SMX) is the most commonly used antibiotic in worldwide for inhibiting aquatic animal diseases. However, the residues of SMX are difficult to eliminate and may enter the food chain, leading to considerable threats on human health. The bacterial strain *Sphingobacterium mizutaii* LLE5 was isolated from activated sludge. This strain could utilize SMX as its sole carbon source and degrade it efficiently. Under optimal degradation conditions (30.8 °C, pH 7.2, and inoculum amount of 3.5 × 10^7^ cfu/mL), *S. mizutaii* LLE5 could degrade 93.87% of 50 mg/L SMX within 7 days. Four intermediate products from the degradation of SMX were identified and a possible degradation pathway based on these findings was proposed. Furthermore, *S. mizutaii* LLE5 could also degrade other sulfonamides. This study is the first report on (1) degradation of SMX and other sulfonamides by *S. mizutaii*, (2) optimization of biodegradation conditions via response surface methodology, and (3) identification of sulfanilamide, 4-aminothiophenol, 5-amino-3-methylisoxazole, and aniline as metabolites in the degradation pathway of SMX in a microorganism. This strain might be useful for the bioremediation of SMX-contaminated environment.

## Introduction

The amount of aquaculture in China ranks first in the world all year round. The huge output mainly depends on high-density aquaculture^1^. This type of aquaculture leads to the risk of outbreaks of aquatic animal diseases. Sulfonamides are synthetic drugs used as anti-microbial, anti-diabetic, diuretic, anticonvulsant, and herbicidal agents. Among which, sulfamethoxazole (SMX) is one of the most prescribed antibiotics worldwide, can kill aquatic pathogens and can effectively inhibit aquatic animal diseases^2–4^. However, SMX cannot be completely utilized and metabolized by aquatic animals because approximately 50% are excreted to the environment without modification. Consequently, SMX has become a widely distributed pollutant in aquatic and domestic waste waters^5^. Numerous studies have indicated that SMX has adverse ecological effects and may threaten human health after long-term exposure. The World Health Organization has classified SMX as a Category 3 carcinogen in 2017^6^. However, SMX has been proven to be difficult to eliminate by conventional water treatment processes^7^. The residues of SMX enter the food chain, leading to considerable threats to human health. Therefore, an efficient and reliable degradation method for removing SMX residue from the water environment must be developed^8^.

SMX removal is mainly by physical, chemical, and microbial methods^9^. The physical and chemical methods mainly include anion exchange, nano-filtration, struvite precipitation, and electrodialysis^10^. However, for technical and economic reasons, physical and chemical techniques may not be feasible^11^. Microbial biodegradation has advantages in terms of its eco-friendliness, low cost, and scope of implementation and has been proven as an effective method for the remediation of SMX residues in aquaculture waters^12^. To date, more than 20 strains of SMX-degrading strains have been isolated from different environments, and all of them exhibit SXM-degrading ability under laboratory conditions. Liang et al. isolated *Achromobacter* sp. JL9, which can use SMX as the sole nitrogen source for growth. The degradation rate of SMX was 90.4%, and the highest reaction rate constant was 0.0384 min^−1^^13^. Gao et al. found that *Phanerochaete chrysosporium* has a strong tolerance to sulfamethoxazole in the concentration range of 10–30 mg/L^14^. At 10 mg/L, the degradation rate reached 53% after 24 h and 74% after 10 days. Jia et al. reported that the sulfate-reducing bacteria (SRB) sludge system shows considerable ability to degrade SMX. When the initial concentration is 25, 50, 100, 150, and 200 mg/ L, the removal rates of SMX by SRB sludge through adsorption and biodegradation are 3.9, 5.6, 13.2, 15.9, and 21.3 mg/L/d^15^. Other bacteria, including *Achromobacter denitrificans* PR1^16^, *P. chrysosporium*^17^, *Pseudomonas* stutzeri^18^, and Acinetobacter sp.^19^, could serve as resources for the bioremediation of SMX from the polluted environment. Nevertheless, further studies are needed. Previous studies mainly isolated SMX-degrading bacteria through traditional methods, and the degradation efficiency was relatively low. The degradation mechanism of SMX and the metabolic pathway involved are also unclear.

The aims of this study were: (1) to isolate a bacterial strain that can highly degrade SMX by a novel method; (2) to optimize the environmental parameters to improve degradation efficiency; and (3) to deduce the possible degradation pathway of degrading strain and examine the mechanism underlying SMX degradation.

## Results

### Community changes and diversity of SMX-degrading enrichment cultures

**Figure 1:**
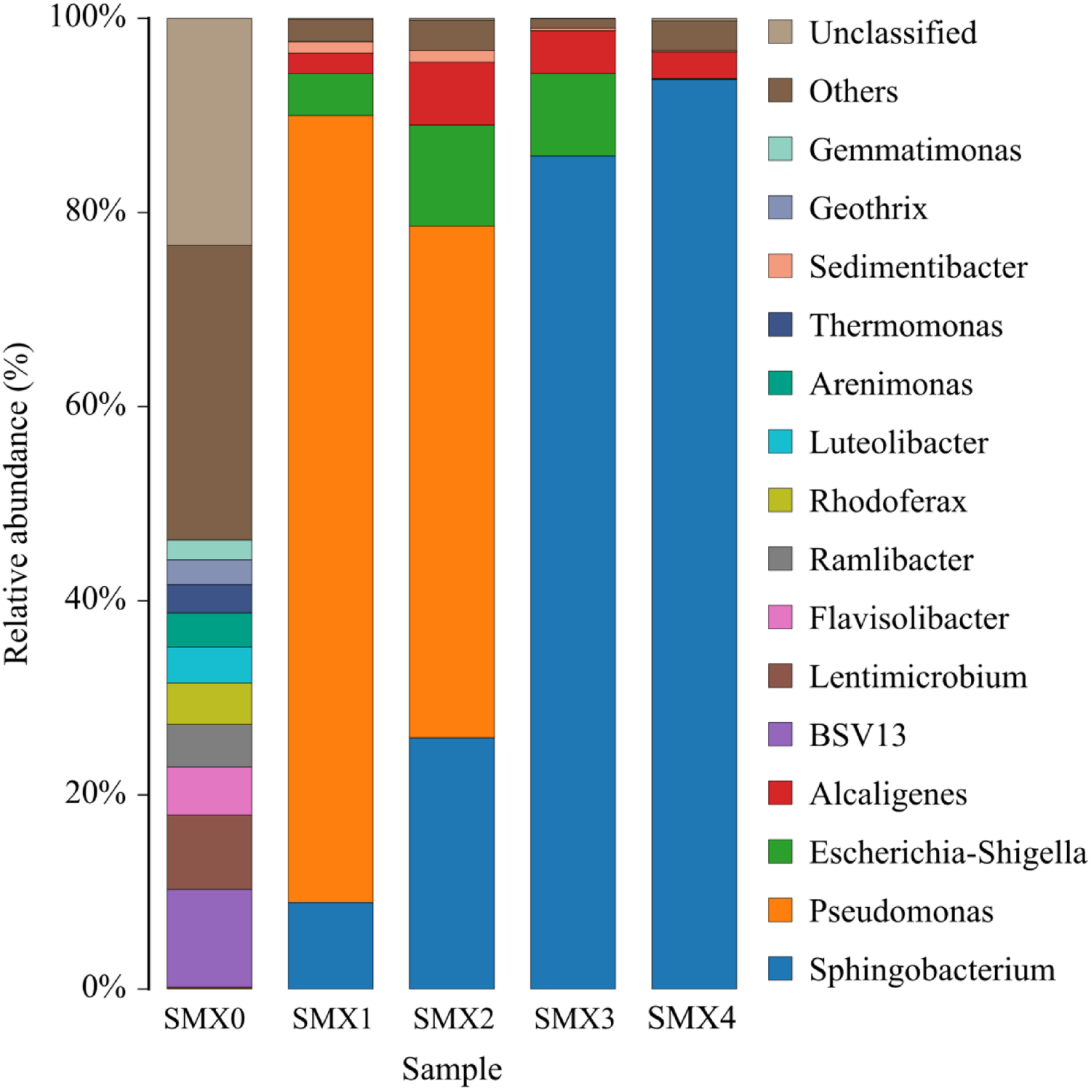
Diversity and community changes in enrichment cultures SMXY, SMX1, SMX2, SMX3 and SMX4 based on the relative abundance of the illumina sequences. The histogram was constructed by using R software.

A total of 5978, 6012, 6048, 6005, and 6003 16S rRNA gene sequences of the distinct V3–V4 regions were obtained from sludge sample (SMXY) and four generations of enrichment cultures (SMX1–4) by high-throughput sequencing, respectively. After statistical analysis and annotation, the results (Fig. 1) showed that 5978 sequences in sludge sample SMXY were clustered into 166 OTUs. The highest abundance of OTUs belonged to bsv13, *Lentimicrobium*, *Flaviolibacter*, *Ramlibacter,* and *Rhodoferax*. After enrichment, the bacterial diversity of SMX1 rapidly decreased, 6012 sequences were clustered into only 23 OTUs. The main genera were *Pseudomonas*, *Sphingobacterium*, *Escherichia*-*Shigella*, *Alcaligenes*, and *Sediminibacter*. *Pseudomonas* became the highest abundance genus in SMX2. After the second passage, with the increase in the SMX concentration, the bacterial diversity of SMX2 decreased continuously, and 6048 sequences were clustered into 20 OTUs. The main genera were *Sphingobacterium*, *Escherichia-Shigella*, *Alcaligenes*, and *Sediminibacter*, *Sphingobacterium* replaced *Pseudomonas* as the genus with the highest abundance, and the proportion of *Sphingobacterium* reached 85.84%. The result indicates that *Sphingobacterium* can tolerate higher concentrations of SMX than the other bacteria. As a result, the diversity in the enrichment gradually declined with the increase in SMX concentrations and passage generations. After the third and fourth passages, the 6003 sequences of SMX4 were clustered into 16 OTUs, and the main genera were *Sphingobacterium*, *Alcaligenes*, and *Sediminibacter.* The proportion of *Sphingobacterium* reached 93.68% and became the dominant genera. This result provides strong evidence that *Sphingobacterium* contributed to SMX degradation. By contrast, the rapid reduction in the proportion of *Pseudomonas* and other bacteria indicates their lack of ability to tolerate high SMX concentrations.

### Isolation and identification of SMX-degrading strains

**Figure 2:**
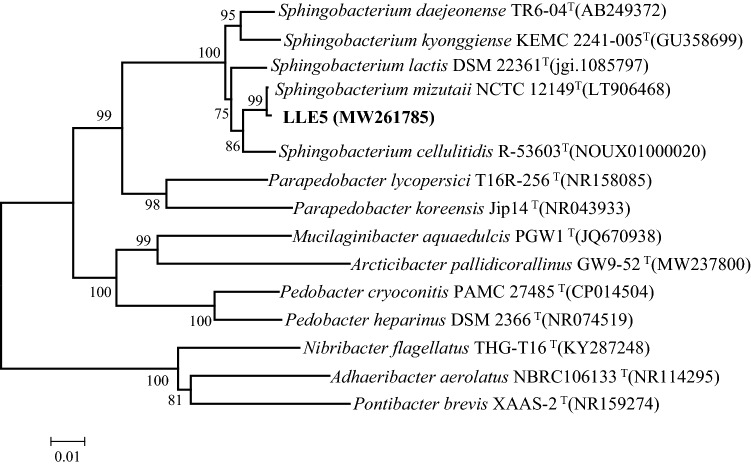
Neighbour-joining phylogenetic tree based on a comparison of the 16S rRNA gene sequences of *S. mizutaii* LLE5 and its closest relatives and several out-group strains. The numbers at the nodes indicate the percentages of bootstrap sampling derived from 1000 replications. GenBank accession numbers are given in parentheses. Bar, 0.01 nucleotide substitution per nucleotide position.

Five strains that showed SMX degradation ability were isolated from SMX4 and were named LLE1–5. Among them, LLE5 utilized SMX as the sole carbon source in MSM and degraded 91.3% of 50 mg/L SMX in 7 days. It was selected for further morphological physiological, and biochemical analyses. LLE5 colonies on LB plate had smooth surfaces and clear edges and were light yellow in color. Under the microscope, cells of LLE5 featured short rods that were 0.6–1.8 μm in length, 0.4–0.7 μm in width, and were gram negative and nonmotile. LLE5 could utilize dextran, D-maltose, D-trehalose, D-fiber, gentian disaccharide, disaccharide, sucrose, D-pinobiose, and stachyose but not D-sorbitol, D-mannitol, and D-arabinol. The 16S rRNA gene sequence of LLE5 was sequenced and submitted to GenBank with the accession number MW261785. Blast results showed that LLE5 belonged to the genus *Sphingobacterium* and clustered strongly with *S. mizutaii* NCTC 12149^ T^ (99.09%, accession number LT906468) (Fig. 2). According to the results of morphological, physiobiochemical, and 16S rRNA evaluations, LLE5 was identified as a member of genus *Sphingobacterium* and named *Sphingobacterium mizutaii*. This is the first report that a *Sphingobacterium* species can degrade SMX. *S. mizutaii* LLE5 has been deposited in Guangdong Microbial Culture Collection of Center under number CGMCC 61038.

### Optimization of the degradation conditions for LLE5

**Figure 3:**
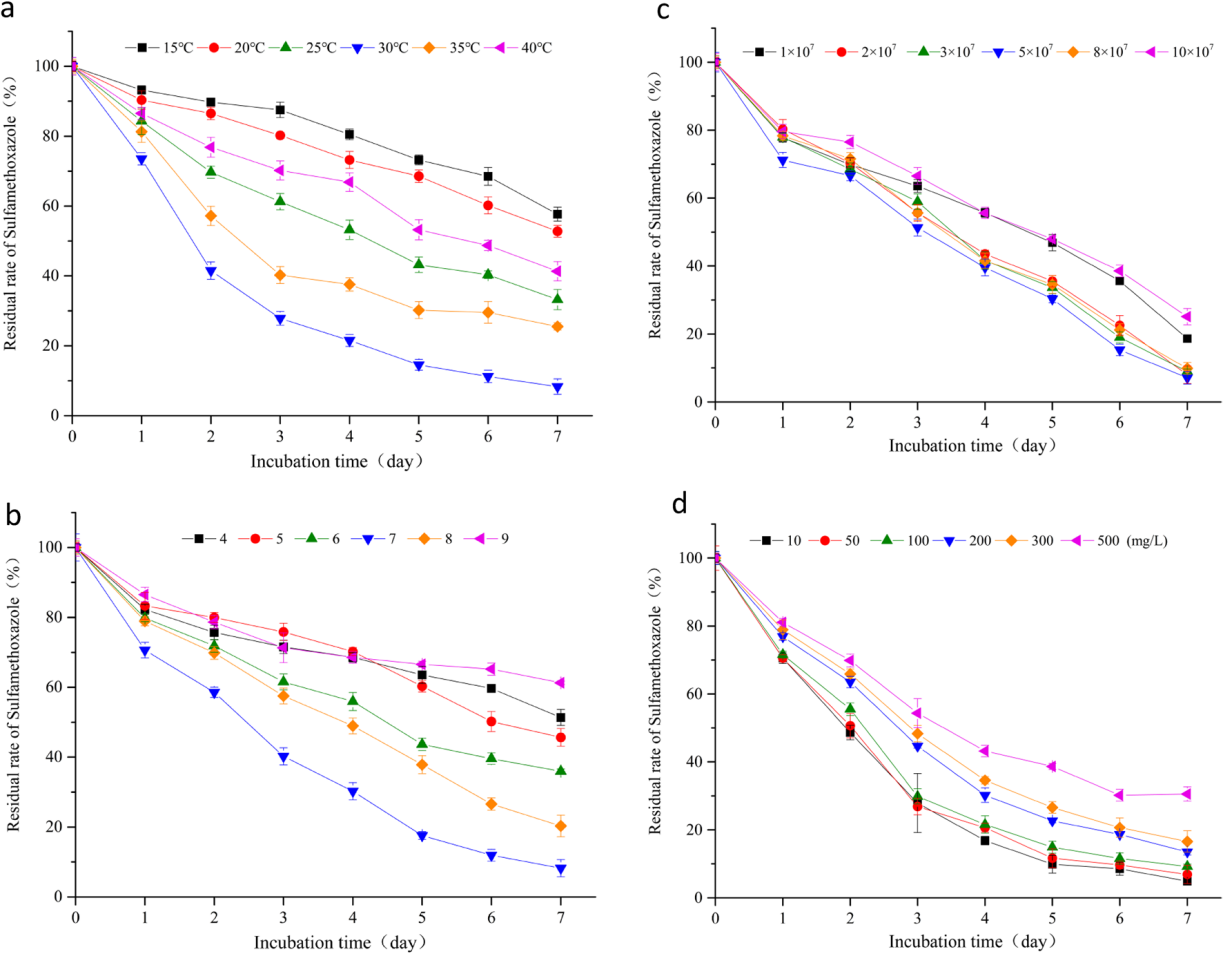
Degradation kinetics of SMX under different conditions (**a**) temperature (°C), (**b**) pH level, (**c**) inoculum amount (× 10^7^ cfu/mL), and (**d**) initial concentration (mg/L) of SMX. The symbols represent averages of triplicate experiments and the error bars indicate their corresponding standard deviations.

The influence of various environmental factors on the biodegradation efficiency was determined. *S. mizutaii* LLE5 can degrade SMX from 15 to 40 °C (Fig. 3a), with an optimum temperature of 30 °C. Although the temperature decreased to 15 °C, the degradation efficiency was more than 42.32%. When the temperature rose to 40 ℃, the degradation efficiency was more than 58.65%, and these results showed that *S. mizutaii* LLE5 has an obvious temperature adaptability. pH is another key factor affecting the degradation efficiency of the strain. The results of different pH on the degradation of the strain (Fig. 3b) show that LLE5 can degrade SMX in the pH range 4.0–9.0. At the optimal pH of 7.0, the degradation of SMX was 91.77%. In addition, the degradation efficiency of LLE5 was more than 50% in the pH range 5.0–8.0, which indicated that the degradation efficiency of strain LLE5 was higher under neutral conditions. When the pH was reduced to 4.0, the degradation efficiency was still 48.64%, indicating that the strain was tolerant to acid conditions. When the pH was 9.0, the degradation efficiency was 38.70%, which indicated that LLE5 could be applied under neutral and acid conditions. The different initial inoculum amount on the degradation efficiency was analyzed (Fig. 3c). When the inoculation amount was 5 × 10^7^ cfu/mL, the degradation efficiency of *S. mizutaii* LLE5 was the highest, which was 92.89%. However, when the initial inoculation amount was 2.0 × 10^7^–8.0 × 10^7^ cfu/mL, the degradation efficiency was more than 90%, indicating that the degradation efficiency of *S. mizutaii* LLE5 did not change significantly with the change in inoculum amount. The initial concentration of SMX also influenced the degradation efficiency of *S. mizutaii* LLE5 (Fig. 3d). The degradation efficiency of *S. mizutaii* LLE5 with 10 mg/L SMX reached 95.14% and decreased to 83.42% when the SMX concentration reached 300 mg/L. These results indicate that high concentrations of SMX inhibit the growth of *S. mizutaii* LLE5.

**Figure 4:**
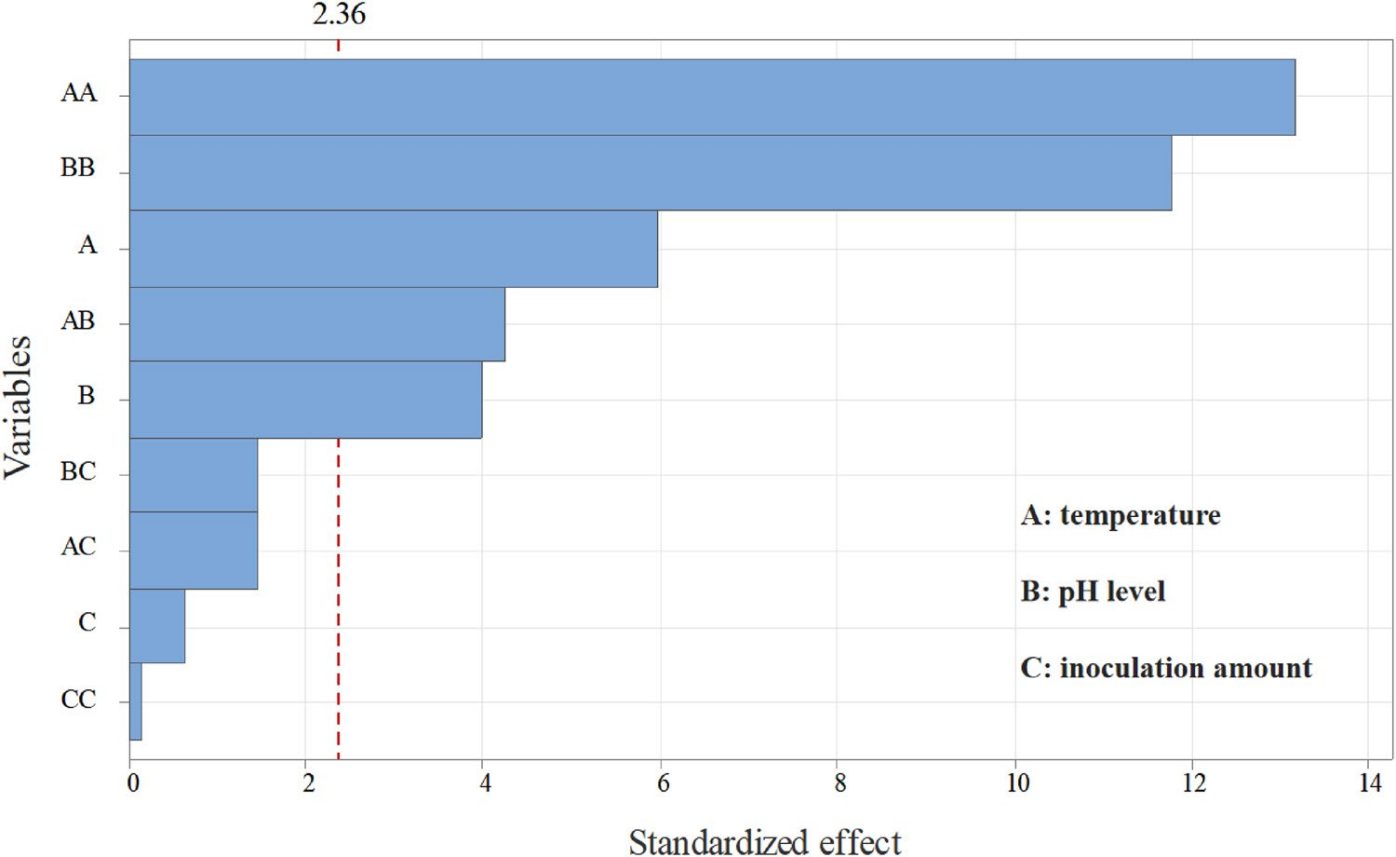
Pareto chart showing the effect of single factor (variables) biodegradation efficiency.

**Figure 5:**
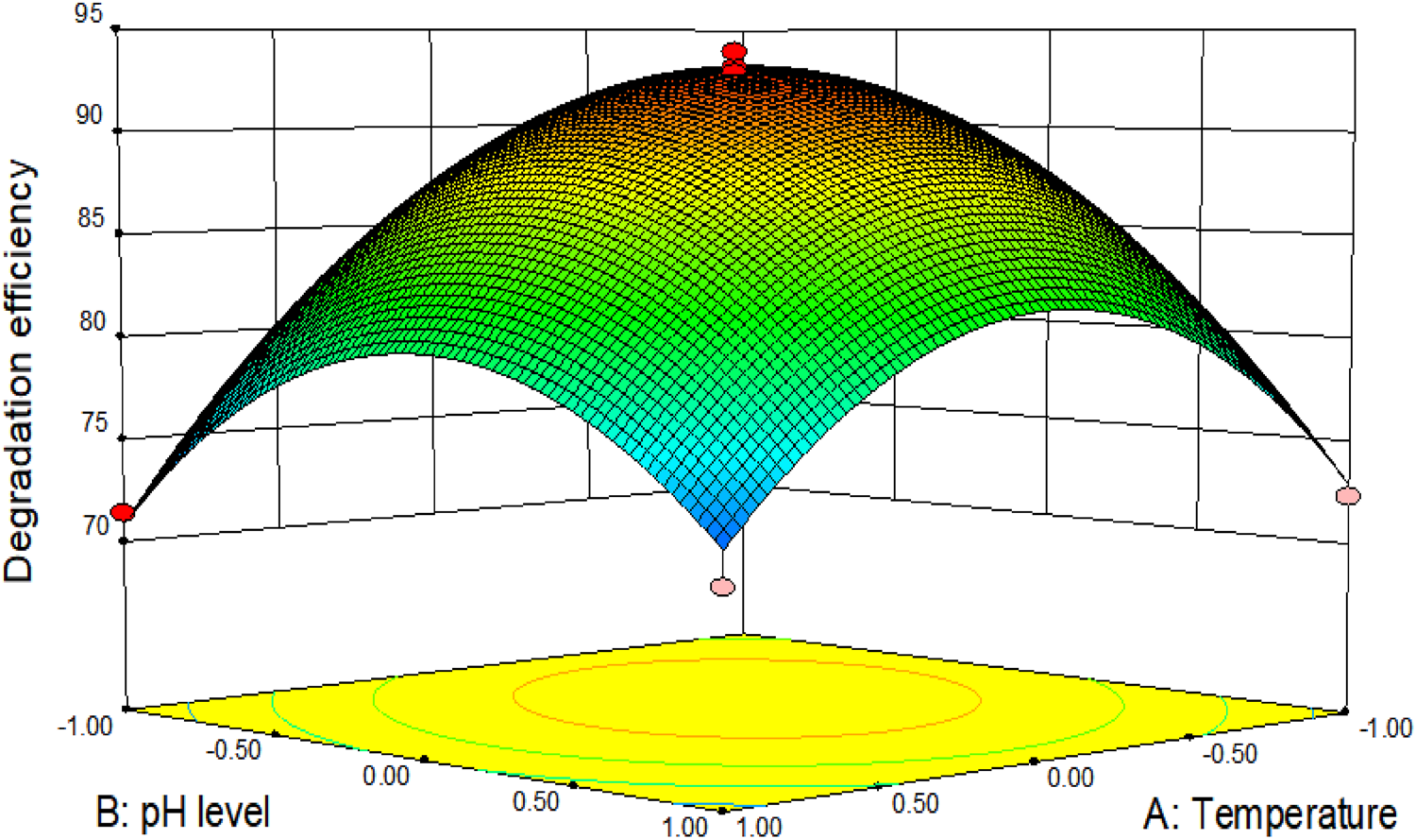
Response surface curves demonstrating the effects of medium temperature (°C) and pH level on SMX biodegradation efficiency (%) with an inoculum amount of *S. mizutaii* LLE5 at 3.5 × 10^7^ cfu/mL.

The temperature, pH levels and inoculation size were further designed through the response surface method in accordance with the results of single-factor experiments, and 17 degradation tests were carried out (Table 1). By the statistical analysis of data, the following second-degree polynomial equation was obtained to explain SMX biodegradation by *S. mizutaii* LLE5:

1 $$ Y_{1} = 92.74{-}3.09A{-}2.06B + 0.32C + 3.10AB{-}1.07AC{-}1.08BC{-}9.40A^{2} {-}8.40B^{2} {-}0.12C^{2} $$

where Y_1_ represents the SMX degradation efficiency, and A, B, and C are the coded values for temperature, pH level, and inoculum amount respectively. Analysis of variance (ANOVA) for the fitted quadratic polynomial model is shown in Table 2. The model was signifcant (*P* < 0.05) with *R*^2^ = 0.9831 and Adj *R*^2^ = 0.9613. The results of regression analysis indicated that A, B, AB, A^2^ and B^2^ are significant model terms, whereas the C, AC, BC, C^2^ are nonsignificant model terms. The same was confirmed from the Pareto chart (Fig. 4) in which higher effects were presented in the upper portion and then progress down to the lower effects. It directly shows that the most import factors influencing biodegradation efficiency were A, B, AB, A^2^ and B^2^. The three-dimensional response surface was plotted to directly display the effects of the temperature and pH level on SMX biodegradation. At the theoretical maximum point of response surface (Fig. 5), the optimum conditions for SMX degradation by *S. mizutaii* LLE5 were 30.8 °C, pH 7.2, and inoculum amount of 3.5 × 10^7^ cfu/mL.

**Table 1:** Box-Behnken experimental design with three independent variables.

Run	X_1_	X_2_	X_3_	Degradation efficiency (%)
1	25	8	3	72.3
2	25	5	3	84.8
3	30	6.5	3	93.11
4	25	6.5	1	84.5
5	25	6.5	5	87.1
6	30	8	5	82.6
7	30	6.5	3	92.84
8	35	5	3	71.4
9	35	6.5	1	81.5
10	30	5	5	86.7
11	30	8	1	83.9
12	30	6.5	3	93.87
13	35	8	3	71.3
14	35	6.5	5	79.8
15	30	6.5	3	92.52
16	30	5	1	83.7
17	30	6.5	3	91.37

X_1_: temperature (°C), X_2_: pH level, X_3_: inoculation amount (× 10^7^ cfu/mL).

**Table 2:** Analysis of variance (ANOVA) for the fitted quadratic polynomial model.

Source	Sum of squares	DF	Mean square	F value	*P* value
X_1_	76.26	1	76.26	35.61	0.0006
X_2_	34.03	1	34.03	15.89	0.0053
X_3_	0.85	1	0.85	0.39	0.5499
X_1_X_2_	38.44	1	38.44	17.95	0.0039
X_1_X_3_	4.62	1	4.62	2.16	0.1852
X_2_X_3_	4.62	1	4.62	2.16	0.1852
X_1_X_1_	371.73	1	371.73	173.57	0.0001
X_2_X_2_	296.81	1	296.81	138.59	0.0011
X_3_X_3_	0.062	1	0.062	0.029	0.8701
Model	869.49	9	96.61	45.11	0.0002
Error	3.35	4	0.84		
Total	884.48	16			

*P* Value < 0.05 indicates the model terms are significant.

### Degradation of SMX by strain LLE5

**Figure 6:**
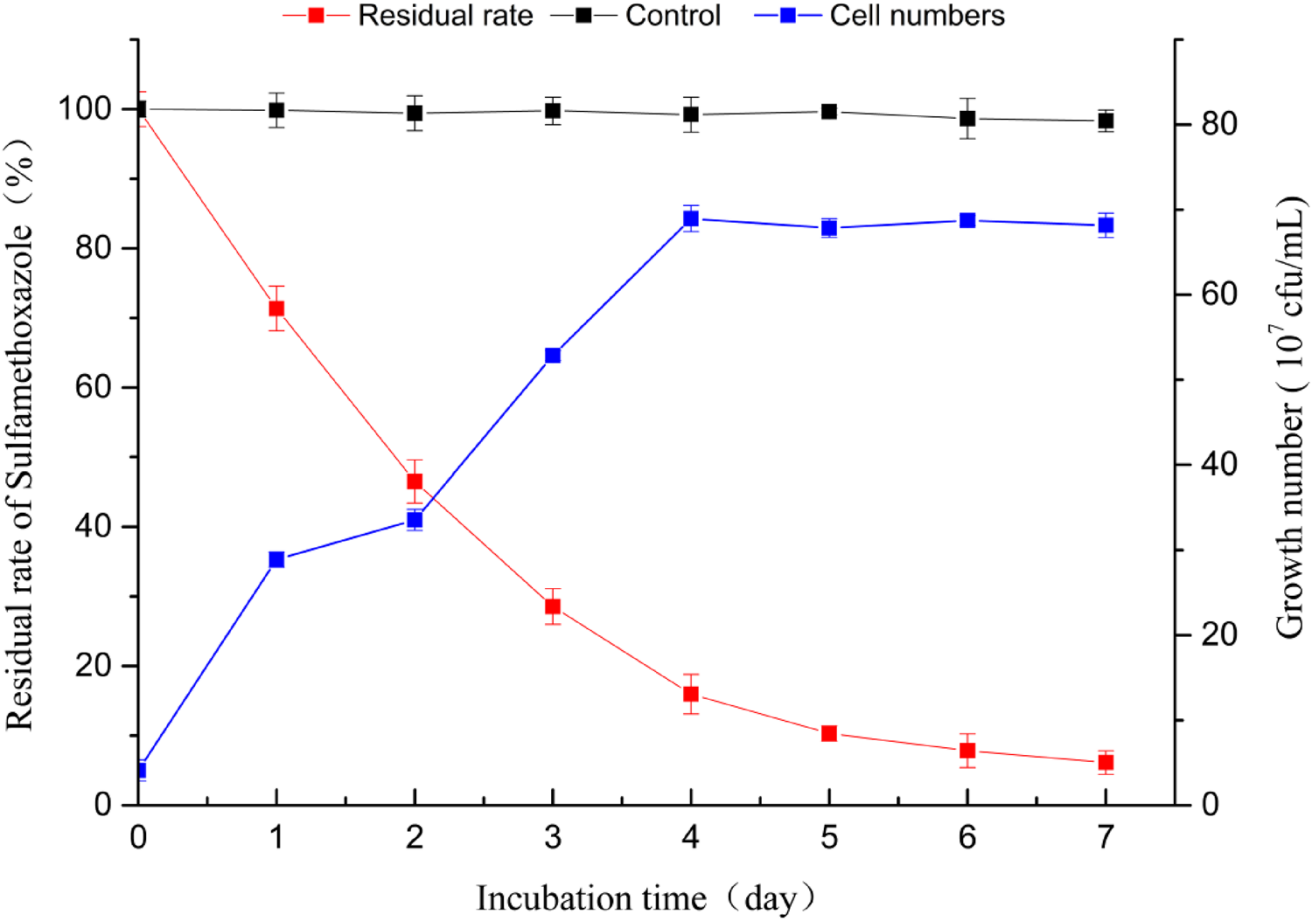
Growth of *S. mizutaii* LLE5 along time and degradation efficiency of SMX. The symbols represent averages of triplicate experiments and the error bars indicate their corresponding standard deviations.

**Figure 7:**
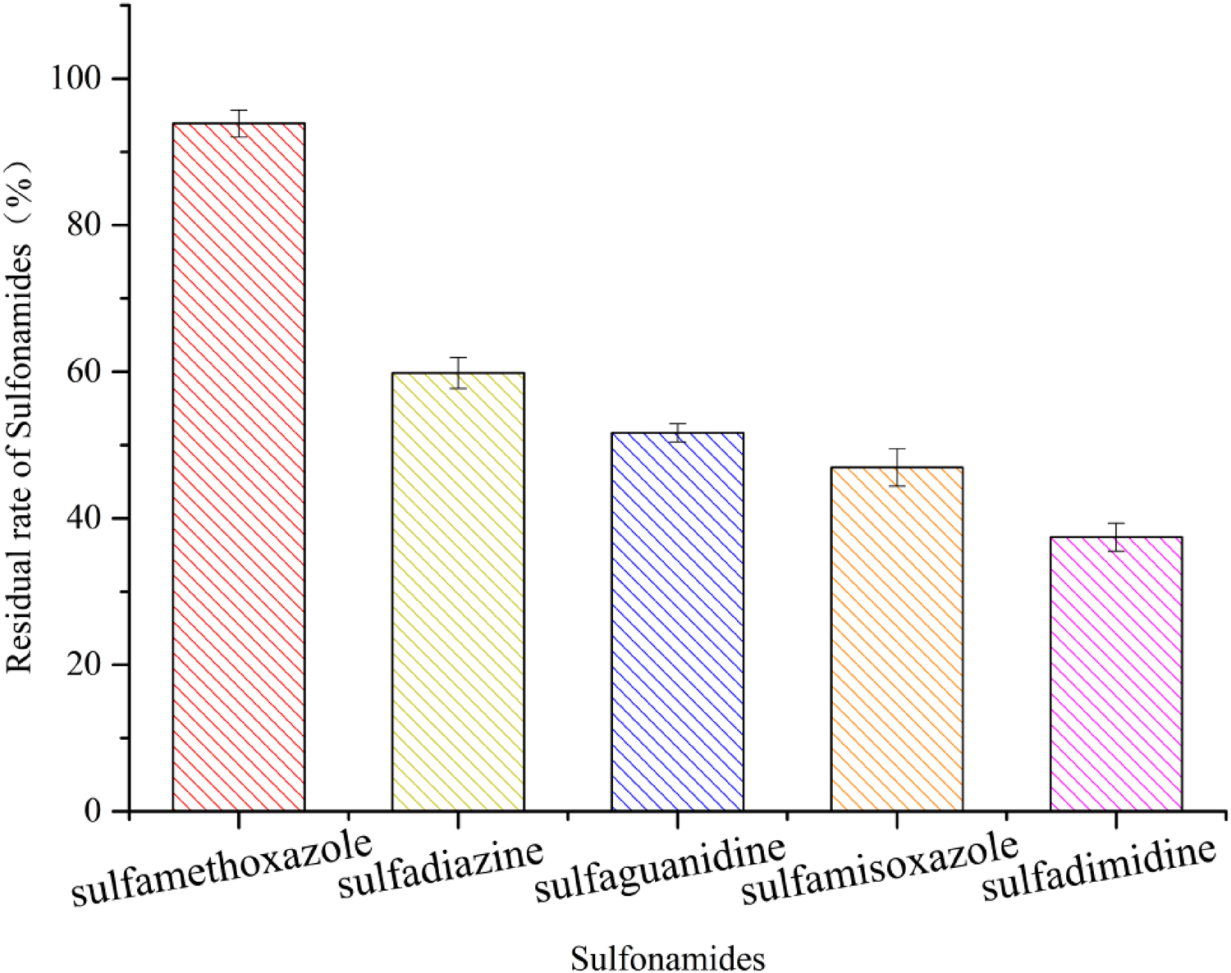
Degradation kinetics of various sulfonamides by *S. mizutaii* LLE5. The symbols represent averages of triplicate experiments and the error bars indicate their corresponding standard deviations.

The degradation characteristics of SMX by *S. mizutaii* LLE5 under the optimal conditions were studied. The results indicated that the most efficient degradation was obtained during the first 3 days. On the third day, the degradation efficiency was 84.02%, and the cell concentration was 68.95% × 10^7^ cfu/mL (Fig. 6). The degradation efficiency was positively correlated with the cell growth density. At 5–7 days, the degradation efficiency of SMX gradually decreased and was accompanied by no further increase in *S. mizutaii* LLE5 cell density. Finally, the degradation efficiency of SMX with initial concentration of 50 mg/L was 93.87% after 7 days. This is the first report that *Sphingobacterium mizutaii* has a good degradation effect on SMX. *S. mizutaii* LLE5 can also degrade other sulfonamides, and the degradation efficiencies of strain LLE5 for sulfadiazine, sulfaguanidine, sulfamisoxazole, and sulfadimidine were 59.85%, 51.68%, 46.95%, and 37.42%, respectively (Fig. 7). To elucidate the degradation ability of *S. mizutaii* LLE-5 against various sulfonamides, the degradation constant (*k*) and half-life (*t*_1*/*2_) were determined by using the first-order kinetic model. Table 3 presents the kinetics parameters calculated from the model. The coefficient of determination *R*^2^ varied from 0.9572 to 0.9924 indicating that the degradation data reliably fitted with the first-order kinetic model. Degradation rate constants (*k*) varied from 0.0620 to 0.4247 d^−1^ that characterized the degradation process of various sulfonamides by strain LLE-5. Theoretical half-life (*t*_1/2_) of SMX, sulfaguanidine, sulfamisoxazole, and sulfadimidine was noted as 1.63, 5.22, 6.80, 7.96, and 11.18 days, respectively. These results show that *S. mizutaii* LLE5 has a broad specificity for the degradation of sulfonamides and has considerable potential for processing sulfonamide pollution in the environment.

**Table 3:** Kinetic parameters of various sulfonamides degradation by *S. mizutaii* LLE-5.

Sulfonamides	Regression equation	k (d^−1^)	t_1/2_ (d)	*R*^2^
Sulfamethoxazole	*C*_*t*_ = 51.3514 × e^−0.4554t^	0.4247	1.63	0.9887
Sulfadiazine	*C*_*t*_ = 50.6351 × e^−0.1327t^	0.1327	5.22	0.9924
Sulfaguanidine	*C*_*t*_ = 50.2014 × e^−0.1019t^	0.1019	6.80	0.9805
Sulfamisoxazole	*C*_*t*_ = 49.8941 × e^−0.0871t^	0.0871	7.96	0.9572
Sulfadimidine	*C*_*t*_ = 50.1347 × e^−0.0620t^	0.0620	11.18	0.9716

k represents degradation constant (d^−1^); t_1/2_ represents half-time (d); *R*^2^ represents correlation coeffificient; Ct is the concentration (mg/L) of sulfonamides at time t.

### Metabolic pathways of SMX degradation by S. mizutaii LLE5

**Figure 8:**
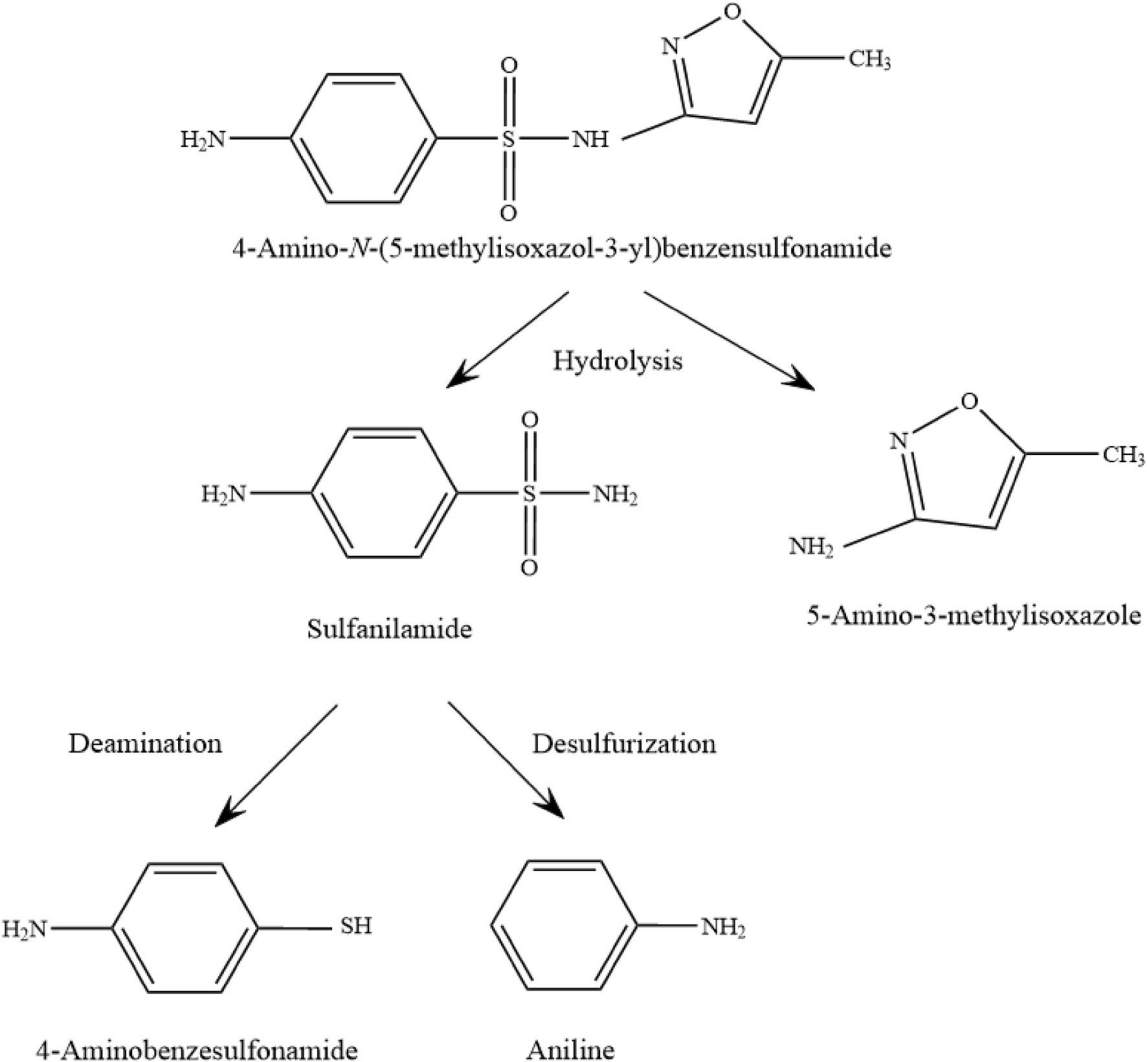
Proposed pathway for the degradation of SMX by *S. mizutaii* LLE5.

The metabolites of SMX degraded by *S. mizutaii* LLE5 in MSM liquid medium were detected by HPLC/MS. According to the chemical structure of SMX and the mass spectrum, four candidate products were identified. These products were sulfanilamide (171 m/z), 4-aminothiophenol (124 m/z), 5-amino-3-methylisoxazole (99 m/z), and aniline (92 m/z). None of these products were detected when the culture medium only contained SMX and without *S. mizutaii* LLE5, indicating that they are SMX biodegradation metabolites. A possible metabolic pathway for SMX biodegradation by LLE5 was proposed (Fig. 8). SMX is first transformed into sulfanilamide and 5-amino-3-methylisoxazole through hydrogenation. Then, sulfanilamide is degraded via desulfurization into aniline and via deamination into 4-aminothiophenol. Although the ring-opening products of hydroquinone were not detected, it is still the first report of the pathway of SMX degradation by a *Sphingobacterium* strain. The candidate products were identified according to their m/z values, SMX chemical properties, and existing reports. Standard samples of four intermediate products were also purchased to conduct parallel experiments.

## Discussion

*Sphingobacterium* strains widely exist in natural environment^20^. *Sphingobacterium* is a kind of gram-negative bacteria and does not produce spores. Since the genus *Sphingobacterium* was proposed originally by Yabuuchi et al. in 1983^21^, new species have been discovered from a variety of environments, such as soil, plants, animals, and even clinical samples of ventricular fluid and urine. Among them, *Sphingobacterum thalpohilum* and *Sphingobacterum multivarum* can degrade petroleum hydrocarbons. For example, *S. multivorum* SWH-2 has a strong ability to degrade petroleum^22^. After ensuring optimal conditions, the normal growth and enzyme secretion and activity of *S. multivorum* SWH-2 will also change, which can further improve the oil degradation of the strain. In addition, most *Sphingobacterium* found thus far are resistant to a variety of antibiotics from microorganisms. However, there is no report on the direct degradation of antibiotics by *Sphingobacterium*. *Sphingobacterium mizutaii* isolated in this study is the first *Sphingobacterium* reported to degrade SMX. The discovery of this bacterium not only expanded the functional range of *Sphingobacterium* but also provided valuable microbial resources for the remediation of SMX contaminated aquaculture environment.

In recent years, a number of strains with good SMX degradation ability, such as *Planococcus kocurii* O516, *Achromobacter* sp. JL9, *Phanerochaete chrysosporium*, and *Acinetobacter* sp., have been isolated from soil, activated sludge, and aquaculture water. Compared with the reported strain, the strain LLE-5 had a better environmental range and tolerance. The study on the influence of environmental factors on the degradation effect indicates that strain LLE-5 can degrade SMX at the temperature range 15–40 ℃ and at pH 4–9. This finding provides a basis for the practical application of the strain in the future. The physiological and biochemical results showed that LLE-5 could use a variety of carbon sources. The bacteria have strong adaptability to the environment, rich types of nutrition metabolism, and potential to degrade other complex compounds.

The existing reports show that most strains of *Sphingobacterium* cannot produce antibiotics but are resistant to antibiotics, which is an important factor for the *Sphingobacterium* to adapt to extreme environments^23^. Antibiotics are compounds that inhibit the growth of microorganisms. Thus, degrading bacteria need to have antibiotic resistance. Some studies have shown that *Klebsiella pneumoniae* can use chloramphenicol as the sole carbon source for growth^24^. However, the drug sensitivity test showed that these strains were sensitive to chloramphenicol, which indicated that the drug resistance and drug degradation processes were two independent pathways. Drug-resistant bacteria can only resist antibiotics and avoid growth inhibition. By contrast, antibiotic-degrading bacteria resist antibiotics and produce degrading enzymes such as monooxygenase, esterase, or hydrolase to degrade the drug. We sequenced the whole genome of strain LLE5 and found some genes that might be involved in SMX degradation (data not shown). Their functions will be identified in our future work. According to the degradation products of SMX by *S. mizutaii* LLE-5, the strain mainly transformed SMX into sulfanilamide and 5-amino-3-methylisoxazole through hydrogenation. Then, sulfanilamide is degraded via desulfurization into aniline and via deamination into 4-aminothiophenol. The results showed that the strain had a clear degradation function of sulfamethoxazole and had a good tolerance. However, SMX could not be completely mineralized in terms of degradation products. This work is the first to report the pathway of SMX degradation by a *Sphingobacterium* strain and provided a certain reference for the study of microbial degradation of SMX.

## Methods

### Chemicals and media

SMX (analytical standard, > 99.5%), HPLC-grade methanol, and acetonitrile were purchased from Sinopharm Chemical Reagent Beijing Co., Ltd. All other reagents used in the present study were of analytical grade. Mineral salt medium (MSM) and Luria–Bertani (LB) medium were used as described by Song et al^25^. Agar plates were prepared by adding 1.5% (w/v) agar into the liquid media.

### Enrichment culture and high-throughput sequencing

Activated sludge samples were collected from the wastewater treatment pond of a pharmaceutical factory in Liaoyang City, Liaoning Province, China. 10.0 g of sample was suspended in 100 mL of MSM supplemented with 100 mg/L SMX. Then, the sample was incubated on a rotary incubator shaker at 30 °C and 100 r/min in the dark. After 7 days, 10 mL of the sample was transferred into fresh MSM supplemented with 200 mg/L of SMX and incubated for another 7 days. After repeating this process four times, the SMX concentration in the fourth generation increased to 500 mg/L. The activated sludge sample was designated as SMXY, and the four generations of SMX enrichment cultures were designated as SMX1, SMX2, SMX3, and SMX4. The total DNA of SMXY and SMX1-4 were extracted using a FastDNA SPIN kit for soil (MP, Biomedicals, USA). After purification, the 16S rRNA distinct regions V3–V4 were amplified with the following primers: 338F (5′-ACTCCTACGGGAGGCAGCA-3′) and 806R (5′-GGACTACHVGGGTWTCTAAT-3′). The polymerase chain reaction (PCR) system was designed as described by Li et al^26^. The PCR product was sent to Biomark Gene Technology Company for library construction and high-throughput sequencing. The obtained sequence data were analyzed using Arch software (http://www.drive5.com/usearch/). The identity of sequences ≥ 97% was assigned to an operational taxonomic unit (OTU), and each OTU was considered to represent a species. The relative abundance of OTUs in the samples was statistically analyzed using R software (https://www.R-project.org)^27^.

### Isolation and identification of SMX-degrading bacteria

According to the results of high-throughput sequencing, the fourth-generation enrichment sample SMX4 containing the most *Sphingobacterium* cells was used for isolation of SMX-degrading bacteria. Then, 100 μL of SMX4 was spread on the MSM solid plate containing 100 mg/L SMX and incubated at 30 °C. The rapidly growing colonies on the plate with different morphologies were selected and restreaked three times to obtain pure cultures. The degradation efficiency of the isolates was determined by a high-performance liquid chromatography (HPLC) system (1260, Agilent, Santa Clara, CA) equipped with an eclipse Plus C_18_ column. 10 mL of the culture and equal volume of ethyl acetate were added into a 50 mL centrifuge tube and mixed using a vortex mixer for 1 min. Then, the centrifuge tube was placed in a shaker, mixed at 220 r/min for 30 min, and centrifuged at 5000 g for 8 min. The upper phase was filtered with a 0.22 μm membrane for HPLC analysis. The elution comprised a mixture of methanol, formic acid (85/15/0.1, v/v/v), and distilled water, running at the flow rate of 1.0 mL/min. The injection volume was 10 μL, and the column temperature was 30°C^28^. The isolate with the highest degradation efficiency was selected for further analysis. Morphology was investigated using a light microscope (BX-51; Olympus, Japan). The carbohydrate assimilation of isolate was conducted with Biolog GEN3 plates following the analytical methods described by Hobbie et al^29^. The genomic DNA of the isolate was extracted and was used as a template for 16S rDNA amplification as described by Ruan et al^30^. The universal primers were 27F (5′-AGAGTTTGATCCTGGCTCAG-3′) and 1492R (5′ACGGHTACCTTGTTTACGACTT-3′). The purified PCR product was sequenced by Bomad Technology (Beijing, China). The obtained sequence was deposited in GenBank. The strain was further identified by performing multiple sequence alignment using Clustal X sofware, and phylogenetic relationships were analyzed via the neighbor-joining (NJ) method with MEGA 6 software^31^.

### Inoculum preparation

Before the degradation experiment, strain activation was performed. *S. mizutaii* LLE-5 was inoculated into 100 mL of LB medium and incubated at 30 °C on a rotary shaker at 150 rpm. After 12 h, the bacterial cells were harvested by centrifugation at 4000 g for 10 min. The precipitate was washed two times by phosphate buffered saline (PBS) solution and suspended for subsequent studies^32^.

### Optimization of the SMX-degrading conditions

The effect of single environmental factors on the biodegradation efficiency was evaluated by analyzing the following parameters and their ranges^33^: temperature (15 ℃, 20 ℃, 25 ℃, 30 ℃, 35 ℃,and 40 ℃); initial medium pH (4, 5, 6, 7, 8, and 9); inoculum amount (1.0 × 10^7^, 2.0 × 10^7^, 3.0 × 10^7^, 5.0 × 10^7^, 8.0 × 10^7^, and 10.0 × 10^7^ cfu/mL); and initial concentration (10, 50, 100, 200, 300 and 500 mg/L). The sterile distilled water was supplemented every 12 h according to the loss of weight. Selected single environmental factors and their interactions were further optimized via response surface methodology (RSM) analysis based on Box − Behnken design^34^. The second order polynomial equation is expressed as follows:

2 $$ Y_{i} = b_{o} + \, \sum b_{i} X_{i} + \, \sum b_{ij} X_{i} X_{j} + \, \sum b_{ii} X_{i}^{2} $$

where *Y*_*i*_ refers to the predicted response, *X*_*i*_ and *X*_*j*_ are variables, *b*_*o*_ is a constant, *b*_*i*_ denotes the linear coefficient, *b*_*ii*_ represents the quadratic coefficient, and *b*_*ij*_ corresponds to the interaction coefficient.

### Biodegradation tests

The SMX degradation test was performed under optimal conditions. *S. mizutaii* LLE-5 cells were inoculated into 100 mL of MSM supplemented with 50 mg/L of SMX. The residual of SMX and cell numbers of *S. mizutaii* LLE-5 were detected every day. The control was inoculated with killed cells of *S. mizutaii* LLE-5. Each test was conducted in triplicate^35^. The ability of *S. mizutaii* LLE-5 to degrade other structurally similar sulfonamides, including sulfadiazine, sulfaguanidine, sulfamisoxazole, and sulfadimidine, were evaluated. Analytical methods were the same as above described. The first-order kinetic model (Eq. 3) was created to elucidate sulfonamides degradation efficiency of *S. mizutaii* LLE-5^36^.

3 $$ C_{t} = C_{0} \times {\text{e}}^{{ - {\text{kt}}}} $$

where *C*_*0*_ is the initial concentration of sulfonamides at time zero, *C*_*t*_ is the concentration of sulfonamides at time *t*, *k* is the degradation rate constant (d^−1^).

The theoretical half-life (*t*_1/2_) values of different sulfonamides were calculated by Eq. (4).

4 $$ t_{1/2} = \frac{{{\text{ln}}\left( 2 \right)}}{k} $$

where ln 2 is the natural logarithm of 2 and *k* is degradation rate constant (d^−1^).

### Identification of SMX biodegradation intermediates

Intermediates generated during SMX degradation by *S. mizutaii* LLE-5 in MSM were analyzed by HPLC–MS (AB Sciex QTRAP 5500, USA). The extraction method was same as described above. The flow rate was 0.2 mL/min, the mobile phase A was 0.1% formic acid (V/V), and B was methanol. The targeted screening gradient elution program was: 0–2 min, 95% A; 2–25 min, 95%–5% A; 25–35 min, 5% A; and 35–40 min, 95% A. The sample injection volume was 20 μL, and the column temperature was 30 °C. The mass spectrometry conditions were: DuoSprayTM ion source, electrospray ionization (ESI), positive ion mode scanning, the ion source temperature was 550 ℃, the spray voltage was 5500 V, and curtain air (CUR) was 35 psi. The accumulation time was 0.25 s, and the collision voltage was (35 ± 15) eV.

## Supplementary Information

Supplementary Information.
