# Author Correction: Biodegradation and metabolic pathway of sulfamethoxazole by *Sphingobacterium mizutaii*

**DOI:** 10.1038/s41598-021-03738-2

**Published:** 2021-12-14

**Authors:** Jinlong Song, Guijie Hao, Lu Liu, Hongyu Zhang, Dongxue Zhao, Xingyang Li, Zhen Yang, Jinhua Xu, Zhiyong Ruan, Yingchun Mu

**Affiliations:** 1 Key Laboratory of Control of Quality and Safety for Aquatic Products (Ministry of Agriculture and Rural Affairs), Chinese Academy of Fishery Sciences, Beijing, 100141 China; 2 Key Laboratory of Healthy Freshwater Aquaculture, Ministry of Agriculture and Rural Affairs, Key Laboratory of Freshwater Aquaculture Genetic and Breeding of Zhejiang Province, Zhejiang Institute of Freshwater Fisheries, Huzhou, 313001 China; 3 College of Food Science and Engineering, Bohai University, Jinzhou, 121013 China; 4 Institute of Agricultural Resources and Regional Planning, CAAS, Beijing, 100081 China

Correction to: *Scientific Reports* 10.1038/s41598-021-02404-x, published online 30 November 2021

The Acknowledgements section in the original version of this Article was incomplete.

“This work was supported by Open Research Fund of Key Laboratory of Healthy Freshwater Aquaculture, Ministry of Agriculture and Rural Affairs (ZJK202101), Central Public-interest Scientific Institution Basal Research Fund, (CAFS, No. 2021A003).”

now reads:

“This work was supported by Open Research Fund of Key Laboratory of Healthy Freshwater Aquaculture, Ministry of Agriculture and Rural Affairs (ZJK202101), Central Public-interest Scientific Institution Basal Research Fund, (CAFS, No. 2021A003) and National Key Research and Development Program of China (No. 2017YFC1600704)."

The original Article has been corrected.

