# Supplementary material for: Biodegradation and metabolic pathway of sulfamethoxazole by Sphingobacterium mizutaii
Source: Sci Rep. 2021 Nov 30;11:23130. doi: 10.1038/s41598-021-02404-x (PMC8632973; doi:10.1038/s41598-021-02404-x)

Fig. S1 The analytical curve of HPLC employed for SMX quantification.


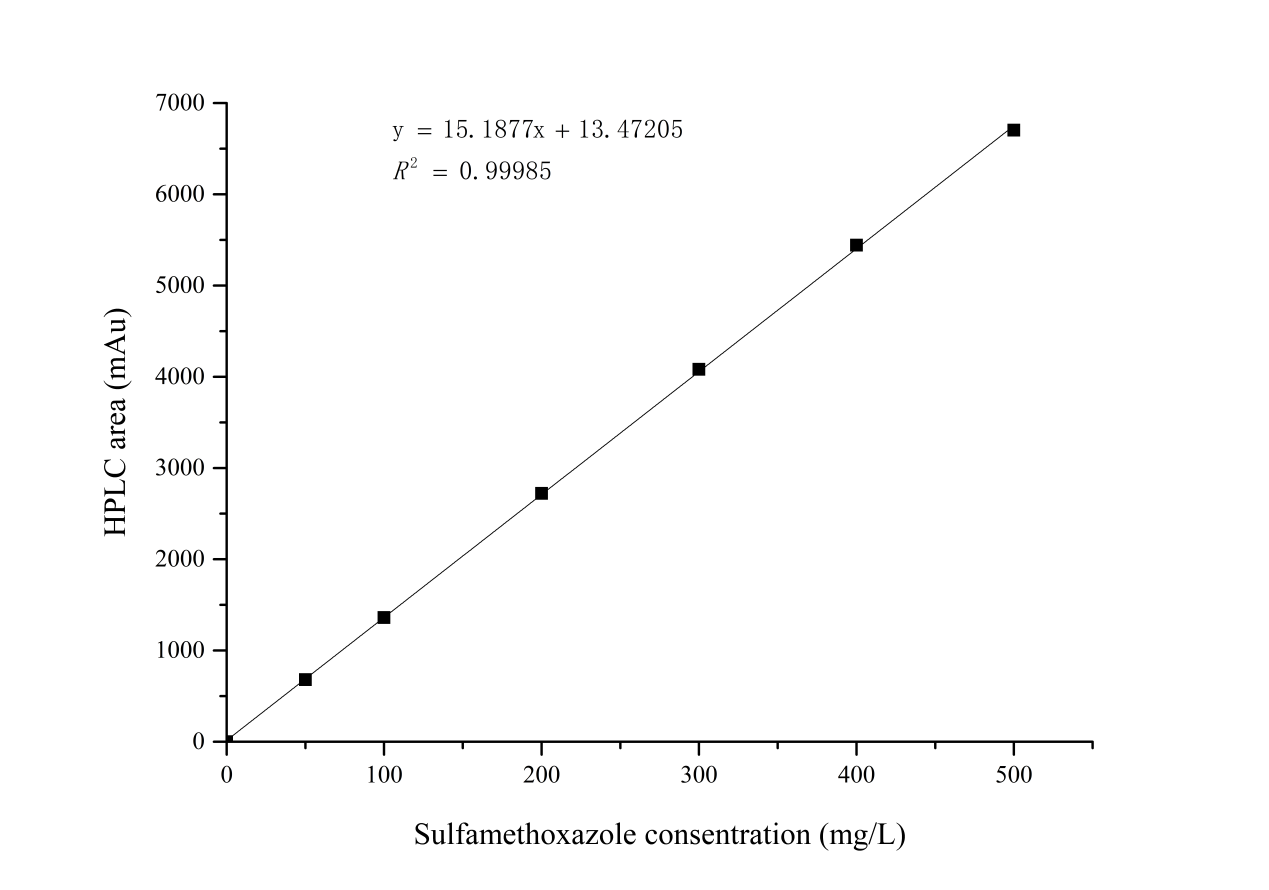


Fig. S2 LC−MS spectrum of intermediates formed during the degradation of SMX by *S. mizutaii* LLE5.

1. LC−MS spectrum of sulfanilamide (171 m/z in negative ion mode ).

1. LC−MS spectrum of 4-aminothiophenol (124 m/z in negative ion mode ).


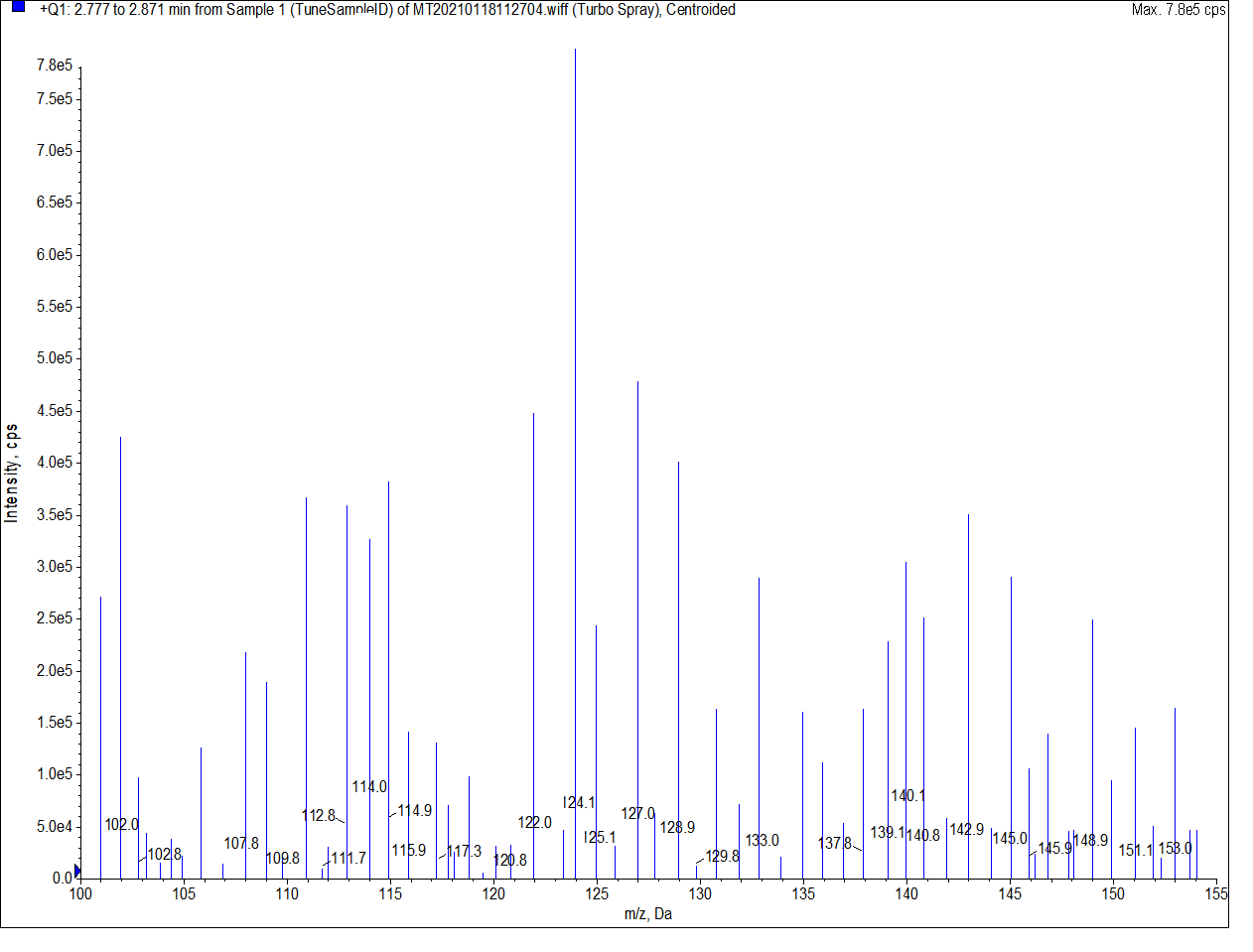


1. LC−MS spectrum of 3-amino-5-methylisoxazole (99 m/z in positive ion mode)

1. LC−MS spectrum of aniline (92 m/z in negative ion mode)


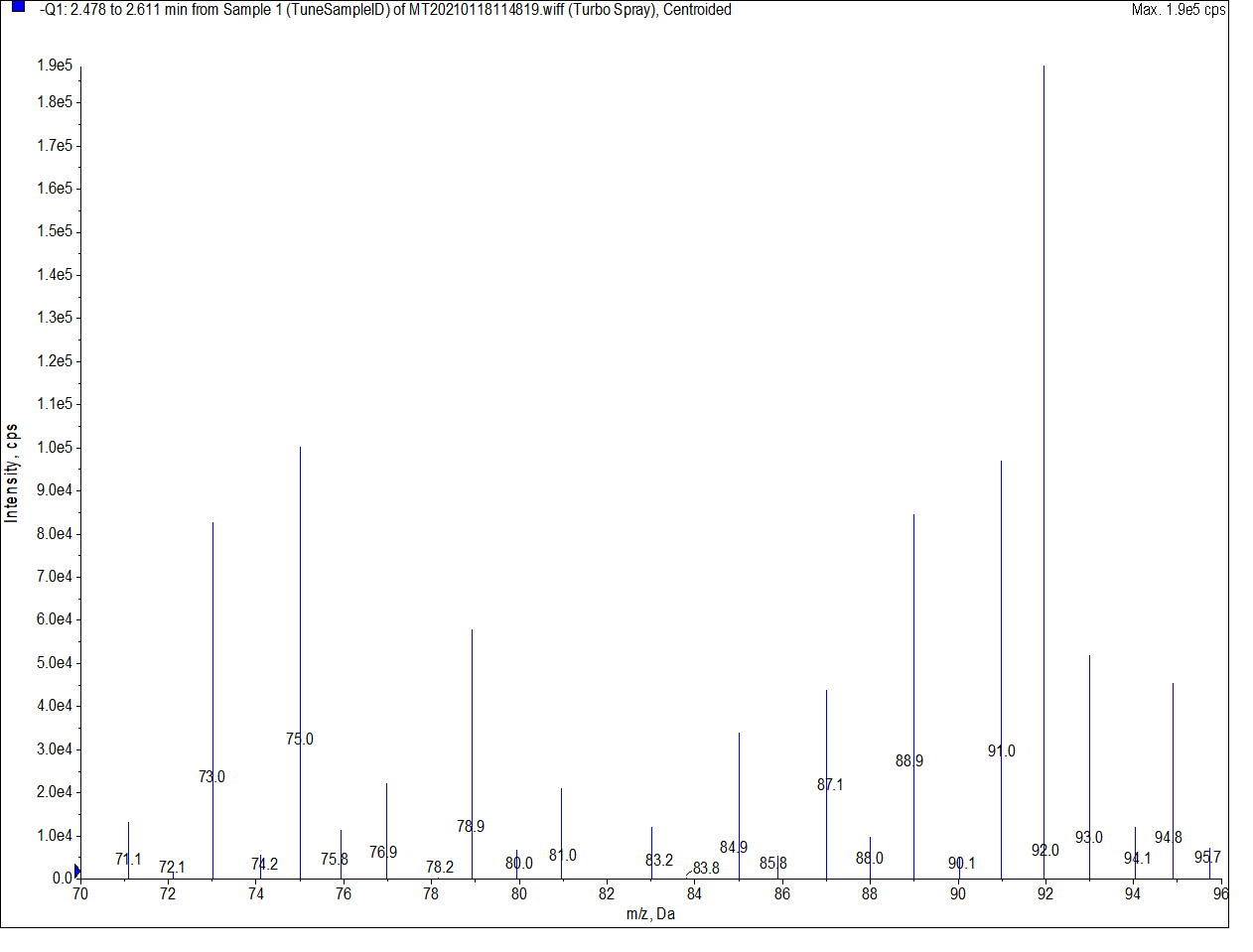

Supplement: Supplementary file 1 — Supplementary Information. [file 41598_2021_2404_MOESM1_ESM.docx]
